# Machine learning models incorporating genotype and ancestry improve severe asthma risk prediction

**DOI:** 10.1038/s41598-025-24080-x

**Published:** 2025-11-17

**Authors:** Nahian Tahmin, Lokesh K Chinthala, Franco Leonel Marsico, Silvia Buonaiuto, Akram Mohammed, Annette Carlisle, Yadu Gautam, Vincenza Colonna, Tesfaye B. Mersha, Robert L Davis, Anahita Khojandi

**Affiliations:** 1 Bredesen Center of Interdisciplinary Research, University of Tennessee, Cumberland Ave, Knoxville, 37996 TN USA; 2 Department of Pediatrics, University of Tennessee Health Science Center, Monroe Avenue, Memphis, 38163 TN USA; 3 Department of Genetics, Genomics and Informatics, University of Tennessee Health Science Center, Monroe Avenue, Memphis, 38163 TN USA; 4 Department of Medicine, Indiana University School of Medicine, Walnut Street, Indianapolis, 46202 IN USA; 5 Department of Industrial and Systems Engineering, University of Tennessee, Cumberland Ave, Knoxville, 37996 TN USA

**Keywords:** Machine learning, Stacking, Genotypic data, Local ancestry, Pharmacogenomics, Asthma, Machine learning, Genome informatics

## Abstract

This study proposes a novel machine learning (ML)-based stacking technique that integrates Single Nucleotide Polymorphisms (SNPs) and inferred local ancestry (LA) to improve predictive accuracy in clinical outcomes. Asthma, particularly severe asthma (SA) with poor response to inhaled corticosteroids (ICS), serves as the case study to illustrate this approach. Using data from the Biorepository and Integrative Genomics (BIG) Initiative, which includes whole-exome sequenced data from a self-reported African American pediatric cohort (N=248), we develop an ML framework to predict ICS response. After SNP data preprocessing and LA estimation, we employ stratified 10-fold cross-validation, creating base pipelines for SNP and LA data, which are then combined in stacked pipelines to assess the effectiveness of integrating these distinct data types. The stacked SNP pipeline yields an AUC of 0.693 ± 0.066 and the stacked LA pipeline yields an AUC of 0.625 ± 0.103. The integration of LA with SNP data significantly improves predictive performance, boosting the AUC to 0.729 ± 0.048 (paired *t*-test *p*-value = 0.005). Pipelines using LA data alone shows comparable performance to those using SNP data alone. However, the most important contributing features are distinct between LA and SNP data demonstrating that these data types capture distinct sources of variation and could provide complementary insights. This study highlights the potential of stacking ML pipelines, based on feature selection techniques and along with logistic regression and random forest predictive models, to integrate SNP and LA data. Such holistic approach has the promise to improve predictive performance of medication response in complex conditions like SA. This approach has broader implications for advancing personalized medicine through the effective use of multifactorial data.

## Introduction

Machine learning (ML) has emerged as a transformative tool across various scientific disciplines, enabling the analysis of complex datasets and the extraction of meaningful patterns^1–3^. These computational advancements are especially pertinent to genetics, where genetic variant data are high-dimensional and sparse and genotype–phenotype relationships can be non-linear, even when sample sizes are relatively limited^4–8^. Thus, ML algorithms are increasingly being adopted in biomedical and genetics research^9,10^, notably for analyzing genetic variant data to provide insights in accelerating drug discovery^11,12^, optimizing clinical decision-making^3,13^, and personalizing treatment plans^14,15^. In particular, a growing body of work applies ML pipelines to uncover associations between genetic variants and disease risk^6,16–18^, demonstrating the potential of ML to capture subtle genotype–phenotype relationships. Despite these advancements, the integration of heterogeneous data in these fields for understanding the treatment outcomes remains underexplored^19–21^. Thus, this manuscript proposes a novel ML technique to leverage both genotype and ancestry data, emphasizing their potential in the genetic underpinnings of complex diseases and treatment strategies.

In genetics research, ML pipelines can process thousands of genetic variants simultaneously selecting the most relevant variants and learning from their genotypic pattern^22,23^. Some ML techniques such as Least Absolute Shrinkage and Selection Operator (Lasso) model and Elastic Net (EN) model are typically used for dimensionality reduction, while other ML models such as logistic regression and Random Forests (RF), along with deep learning methods, have been effectively employed to predict disease risk and treatment outcomes^3,24,25^. Furthermore, ML techniques facilitate the creation of pipelines for multifactorial data types, allowing for extensive analysis by integrating genotypic data with other factors of the disease such as sociodemography and environmental exposures^26,27^. For instance, ensemble methods, like stacking, combine multiple models to enhance predictive accuracy and robustness, effectively integrating multifactorial data and reducing overfitting in small datasets^28,29^. Such ensemble methods are particularly useful in understanding complex diseases and their treatment outcomes.

Here, we focus on asthma, a chronic inflammatory disease marked by airway hyper-responsiveness and variable airflow obstruction^30^, as our case study for multifactorial ML analysis due to its complex genetic underpinnings^31,32^. Despite significant advancements in asthma management, asthma remains quite complex and at times difficult to manage for clinicians due to its heterogeneity in clinical presentation and varied treatment response among patients to mainstay therapies such as inhaled corticosteroids (ICS)^33–35^. While the majority of individuals with asthma benefit from ICS, approximately one-third experience little to no response^36–38^ resulting in severe asthma (SA). Thus, effectively managing asthma patients’ treatment outcomes requires a holistic approach - one that relies on analyzing the genotype as well as the specific genetic constitution of ancestry of the individual that may influence their response to treatment.

In this study, we leverage genotype biomarkers, specifically Single Nucleotide Polymorphisms (SNP), the genetic makeup of an individual including the complete set of genes and genetic variations they possess. Ancestry provides insights into SNP variations, such as differences in disease allele frequencies and linkage disequilibrium (LD) patterns^39–41^. Including ancestry pipelines can adjust the framework for the prevalence of certain genetic variants associated with ICS response in specific ancestral groups^42,43^. We acknowledge that genetic differences alone do not account for health disparities, as social drivers like socioeconomic status, healthcare access, and environmental factors play an important role in health outcomes^44^. However, in this study, our goal is to develop a holistic framework centered on SNP data analysis and to highlight the potential of extracting distinct signals from both genotype and ancestry for improving pipeline prediction performance, an area that remains underexplored.

It is important to note that ancestry refers to an individual’s genetic lineage and geographic origins, derived from biological and genetic markers^45^. It outlines the history of human populations and can be studied scientifically to understand patterns of genetic variation, such as allele frequencies or evolutionary adaptations^45^. Furthermore, the global ancestry measure reflects an individual’s overall genetic background, while the local ancestry (LA)^46–49^ measure identifies the specific ancestral origins of individual genome segments, offering more detailed inheritance insights into specific parts of the genome. In contrast, race is a socially constructed concept based on physical characteristics and is shaped by sociopolitical factors rather than biology. It is worth mentioning that while race may correlate with certain genetic markers due to historical population structures, it is mainly tied to social drivers such as discrimination, socioeconomic status, and environmental exposures, making it more reflective of social experiences than genetics^50^. Ancestry can sometimes serve as a proxy for social and environmental factors because individuals with shared ancestry often experience similar societal conditions^44,51^. In our study, we consider race during patient selection to account for the social context influencing asthma outcomes; however, we use LA as a feature to adjust for biological differences.

Hence, here we focus on SNPs along with LA to explore the biological predictors of ICS response, an underexamined area in comparison to social factors, acknowledging that non-genomic data such as social drivers of health to capture the broader context of risk factors is important but beyond this study’s scope. Our study seeks to investigate and interpret the SNP variations within the context of ancestry providing deeper insights into asthma management.

Past studies have shown the potential of ML techniques in predicting asthma severity and treatment outcomes by integrating clinical^52,53^, environmental^27,54^, and genetic biomarkers data^53,55^. Traditional techniques like RF combined with Support Vector Machines (SVM) have been effective in asthma risk prediction^56^, and deep learning methods like Convolutional Neural Networks (CNN)^57^ and time-sensitive, attentive neural network model^58^ have excelled in SA exacerbation predictions. However, previous studies applying ML to genetic data rarely consider ancestry resulting in ML models with confounding results. To the best of our knowledge, only a few studies^26,59,60^ included global ancestry in their analyses. Even then, global ancestry may not explain risk at specific genomic positions^59^. Indeed, recent literature emphasize the importance of incorporating LA in genomic studies^26,40^ and call for novel ML techniques that integrate various data factors for explainable risk scores in clinical practice^61,62^.

To the best of our knowledge, this is the first study to propose stacked ML pipelines to integrate SNP and LA features and to determine their respective and combined association effect for complex diseases such as asthma. We develop base pipelines that include ML feature selection to identify key SNP and LA features and train the respective predictive models. These pipelines are then stacked and integrated to assess the combined predictive power of the distinct data types in distinguishing the ICS responders from non-responders. This approach highlights the potential of ML to revolutionize asthma management by integrating SNP and LA data for more personalized treatment strategies.

## Methods

### Data description

This study leverages datasets from the Biorepository and Integrative Genomics (BIG) Initiative, made possible through the Genomic Information Commons (GIC) Consortium^63^. The BIG Initiative is a collaborative effort by the University of Tennessee Health Science Center (UTHSC), Le Bonheur Children’s Hospital, and Regional One Hospital, dedicated to collecting a comprehensive repository of Deoxyribonucleic Acid (DNA) samples. The BIG Initiative, launched in October 2015, has enrolled over 31,000 participants from Tennessee, with a goal to collect more samples from clinical partners statewide. The research protocol was approved by UTHSC Institutional Review Board (IRB # 22-09164-NHSR) and all research was carried out in accordance with relevant guidelines and regulations. Informed consent was obtained from all participants and/or their legal guardians, and all research was carried out in accordance with the Declaration of Helsinki.

**Fig. 1:**
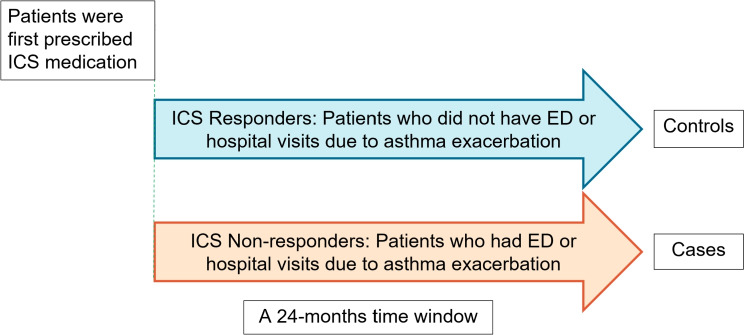
The definition of cases and controls with respect to their ICS response during the first 24 months from their first asthma diagnosis.

Our analysis focuses on a subset of this cohort, specifically 248 participants diagnosed with asthma, filtered based on the following inclusion criteria - asthma diagnosis indicated by International Classification of Diseases (ICD) codes (493.x or J45.x); self-reported African-American (AA); six years of age or older; prescribed ICS; availability of genomics data, specifically whole exome sequencing (WES) data.

Participants are categorized into cases and controls (cases = controls = 124) based on their ICS response. Specifically, cases are ICS non-responders and controls are ICS responders (see Fig. 1). As seen in the figure, cases are patients who experienced emergency department (ED) or hospital visits due to asthma exacerbation in the 24 months following their initial prescription of ICS. Controls are patients who, after being prescribed ICS, did not require ED or hospital visits for asthma exacerbation. Note that to achieve a balanced dataset, we also augment the controls by including patients with the exemption of ICD coded asthma diagnosis. In pediatrics, ICS is exclusively used for moderate-to-severe asthma management^64,65^, suggesting these additional controls may have had asthma diagnoses prior to the coverage of our dataset.

**Table 1:** Demographic information of the study population consisting of self-reported African-American participants.

Characteristics	Overall	Cases	Controls
Patients, n(%)	248 (100)	124 (50)	124 (50)
Female, n(%)	122 (100)	61 (50)	61 (50)
Age (yr.) median (IQR)	16 (13-18)	16 (14-18)	15 (12-18)

Table 1 presents the demographic information of the study population. As seen in the table, 122 (50%) of the participants are female, with cases having a slightly higher median age (16 years, IQR: 14-18) compared with controls (15 years, IQR: 12-18).

#### Genotyping

All samples are sequenced on an Illumina NovaSeq 6000 system on S4 flow cells sequencer using $2\times 75$ paired-end sequencing. All samples are aligned by the Burrows-Wheeler Aligner (BWA) MEM to the GRCh38 assembly of the human reference genome in an alt-aware manner. Duplicates are marked using Picard, and mapped reads are sorted using sambamba. DeepVariant v0.10.0 with a custom exome model is used for variant calling, and the GLnexus v1.2.6 tool is used for joint variant calling.

#### Local ancestry inference

The LA data is first phased, a critical step for accurately determining the ancestral origins of genomic segments. The SHAPEIT software^66^ is used for phasing, aligning haplotypes by inferring the most likely sequence of genetic variants along each chromosome. The phased data is run through RFMix v2 with AFR and EUR superpopulation reference panels from the 1000 Genome Project^67^ for LA inference, simultaneously assigning ancestry to genomic segments and inferring global ancestry based on the reference panels. The included AFR populations are GWD (n=280), YRI (n=187) and MSL (n=128) and the EUR populations are CEU (n=184) and TSI (n=112). The algorithm employs a RF model trained on reference populations to predict ancestry at each genomic segment, providing high-resolution ancestry assignments.

### Machine learning modeling

**Fig. 2:**
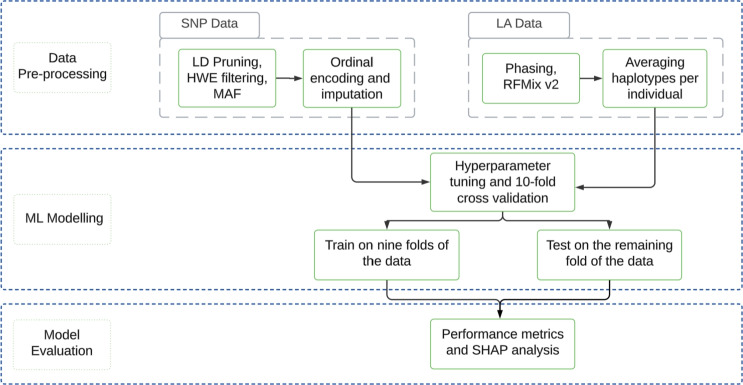
The overview of the methodology for developing the base pipelines—this methodology comprises preprocessing, modeling, and evaluating respective ML models for SNP and LA data. SNP data undergoes LD pruning, HWE filtering, and MAF thresholds, followed by ordinal encoding and imputation, while LA data is processed with phasing and RFMix v2, averaging haplotypes per individual. After preprocessing, nine out of ten folds of the data are used for training and the remaining fold for testing, with hyperparameter tuning and 10-fold cross-validation. Final evaluation includes performance metrics and SHAP analysis to assess feature importance and model interpretability.

In this study, we develop three distinct pipelines to differentiate the cases from the controls using SNP data alone, LA data alone, and their combination. Each pipeline incorporates ML modeling, its evaluation, and interpretation to ensure robust and reliable results. Specifically, we apply ML feature selection techniques to select key SNP and LA features and train separate predictive ML models for these distinct data types, resulting in the base pipelines for our framework. Figure 2 presents the methodology for developing the base pipelines. This proposed methodology comprises three key stages, namely, data preprocessing, ML modeling, and model evaluation. Next, the respective pipelines for the SNP and LA data are stacked to assess the predictive signals for each data type. These stacked pipelines are then integrated, allowing us to evaluate the combined predictive power of both SNP and LA data in distinguishing ICS responders from non-responders.

#### SNP data preprocessing

To ensure comprehensive data filtering, domain specific preprocessing steps are undertaken using PLINK software^68,69^. The Minor Allele Frequency (MAF) filtering step involves applying a minor allele frequency threshold (MAF < 5%), which removes SNPs with low MAF. Similarly, Hardy-Weinberg Equilibrium (HWE) filtering step requires applying Hardy-Weinberg Equilibrium (alpha = 0.05). Finally, Linkage Disequilibrium (LD) pruning (window size = 50 kb, step size = 5, $\hbox {r}^{2}$ = 0.2), performed using the PLINK software, selects a subset of SNPs that are not highly correlated, reducing redundancy and multicollinearity in the dataset.

After this data preprocessing using PLINK, ordinal encoding is used to transform the categorical SNP information into numerical format suitable for ML algorithms. SNP data, initially represented as ‘./.’, ‘0/0’, ‘0/1’ or ‘1/0’ and ‘1/1’, are encoded as 0, 1, 2, and 3 respectively. This transformation allows the ML models to process the SNP data as numerical inputs, with a special emphasis on the prevalence of mutations. Missing SNPs (approximately 2%), encoded as 0, are imputed using KNNImputer by taking the average of the five (n=5) closest SNP values. With this final step, the dataset consists of only 1, 2, and 3 encoded SNP data values.

#### LA data preprocessing

For data resulting from RFMix v2^46^, the LA assignments (1 if AFR LA code and 0 otherwise) are averaged across the two haplotypes for every individual, resulting in a single LA value for each genomic segment. This provides a summary LA measure for each segment, reducing dimensionality and making the data amenable to ML modeling. Consequently, the LA assignments for each segment are matched to all the SNPs located at the segment and these SNPs are assigned the same LA code as its corresponding segment.

#### Stacking

**Fig. 3:**
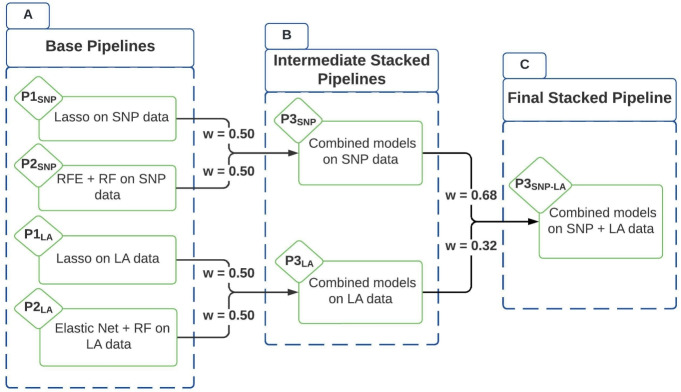
The overview of the proposed ML stacking framework—the framework stacks the base ML models and develops intermediate pipelines $P3_{SNP}$ and $P3_{LA}$ and consequently the final stacked pipeline $P3_{SNP-LA}$ to achieve improved prediction performance of the clinical outcome of interest through leveraging both SNP and LA data.

The model training phase involves developing ML models that can accurately differentiate ICS responders from non-responders based on SNP and LA data. To minimize overfitting and maximize generalizability, we tune, train and evaluate our pipelines applying 10-fold cross-validation on the preprocessed data as shown in Fig. 2. This procedure yields the base pipelines, which are then stacked into intermediate and final pipelines as depicted in Fig. 3.

The first SNP data pipeline ($P1_{SNP}$) employs Lasso model, a linear model that uses L1 regularization to select a sparse set of predictive features, as seen in Fig. 3. This method ensures that only the most linearly relevant SNPs are included in the model. The second SNP data pipeline ($P2_{SNP}$) consists of Recursive Feature Elimination (RFE) followed by RF model. In each fold, RFE iteratively removes the least important features based on their importance scores derived from the RF model. The RF model, with its ensemble of decision trees, captures complex, non-linear interactions among SNPs and is robust to overfitting.

Similarly, the first LA data pipeline ($P1_{LA}$) uses Lasso model to identify key SNP with LA signals linearly associative with ICS response. The second LA data pipeline ($P2_{LA}$) employs EN model followed by an RF model. EN combines L1 and L2 regularization, balancing feature selection and regularization. This approach mitigates the limitations of Lasso model, which may select only one of several correlated features. The selected features are then used in an RF model. At the *P*1 stages, the priority is low-variance, high-interpretability linear feature screening for downstream classification. In *P*2 stages, LA segments exhibit tract-level block correlation, so $P2_{LA}$ retains correlated groups while regularizing; SNPs are ultra–high-dimensional with complex interactions, so $P2_{SNP}$ conducts redundancy pruning guided by the RF’s importance scores. For each pipeline, hyperparameter tuning is done on each fold using cross-validation to select the optimal hyperparameters, ensuring that the model balances complexity and predictive performance.

The next stage of the methodology involves integrating pipelines through a stacking approach, combining the strengths of individual pipelines to enhance predictive performance while balancing their potential shortcomings. Intermediate stacked pipelines, depicted in Fig. 3, are first created for SNP and LA data. The intermediate SNP stacked pipeline ($P3_{SNP}$) combines the prediction probabilities of the two base SNP pipelines ($P1_{SNP}$ and $P2_{SNP}$) with equal weighting. Similarly, the intermediate LA stacked pipeline ($P3_{LA}$) combines the prediction probabilities of the two base LA pipelines ($P1_{LA}$ and $P2_{LA}$) with equal weighting. When stacking probability outputs, a decision threshold converts continuous probabilities into categorical labels; in our approach, the probability threshold of 0.50 is used for both pipelines. This intermediate stacking approach enhances robustness by leveraging the strengths of both models, capturing the linear and non-linear associations.

As seen in Fig. 3, the final stacked pipeline ($P3_{SNP-LA}$) are obtained by integrating the intermediate SNP ($P3_{SNP}$) and LA ($P3_{LA}$) stacked pipelines. Weighted probabilities of 0.68 and 0.32 are used for $P3_{SNP}$ and $P3_{LA}$ respectively and probability threshold at 0.52, determined through cross-validation tuning to optimize performance. These weights and threshold are selected by evaluating candidate SNP/LA weight pairs and thresholds within the same stratified 10-fold cross-validation and choosing the values that maximized average predictive performance across folds. This final step aims to balance the contributions of SNP and LA data, providing a more holistic pipeline that captured the multifactorial nature of ICS response and enhancing the overall predictive performance of the proposed framework.

Finally, we run SHapley Additive exPlanations (SHAP) on each fold of $P1_{SNP}$, $P1_{LA}$, $P2_{SNP}$, and $P2_{LA}$ to better interpret our results. For each base pipeline, the SHAP values are averaged across the folds and the loci with non-zero SHAP values in eight or more folds are filtered to identify the most important loci from each pipeline.

## Results

In this section, we present the performance of the pipelines using 10 folds cross-validation ensuring robust evaluation of our framework. In each fold, the testing set consisted of 25 patients and the rest of the data is used for training. The performance of the pipelines is evaluated using the metrics - area under the receiver operating characteristic curve (AUC), weighted precision, weighted recall, weighted specificity, and balanced accuracy - averaged across 10 folds. We opt for weighted metrics evaluation to offer a holistic view of performance across cases and controls as it averages the metrics of both classes equally. Consequently, a comparison between P1 and P2 pipelines with their respective models assesses the power of stacking two different ML modeling for the same feature. Additionally, contrasting P2 pipelines to P3 pipeline evaluates the predictive capability of integrating two distinct feature types.

**Table 2:** Comparison of performance metrics across the base pipelines.

Metric	SNP base pipelines		LA base pipelines	
	$P1_{SNP}$	$P2_{SNP}$	$P1_{LA}$	$P2_{LA}$
AUC	0.678 ± 0.091	0.641 ± 0.072	0.602 ± 0.103	0.605 ± 0.108
Weighted precision	0.647 ± 0.062	0.643 ± 0.071	0.591 ± 0.103	0.578 ± 0.089
Weighted recall	0.642 ± 0.059	0.635 ± 0.066	0.586 ± 0.099	0.575 ± 0.084
Weighted specificity	0.638 ± 0.062	0.631 ± 0.064	0.589 ± 0.100	0.573 ± 0.088
Balanced accuracy	0.640 ± 0.060	0.633 ± 0.064	0.588 ± 0.100	0.574 ± 0.086

Values after the plus-minus sign represent standard deviation (SD)

Table 2 presents the stable performance of the SNP pipelines, with $P1_{SNP}$ and $P2_{SNP}$ pipelines achieving mean AUCs of 0.678 and 0.641 respectively. In contrast to SNP pipelines, the $P1_{LA}$ and $P2_{LA}$ pipelines achieves slightly lower mean AUCs of 0.602 and 0.605 respectively. Next, Table 3 presents the performance of the stacked $P3_{SNP}$ pipeline achieving an improved mean AUC of 0.693 in comparison to its base pipelines as presented in Table 2. Similarly, the stacked $P3_{LA}$ pipeline achieves an improved mean AUC of 0.625 in comparison to its base pipelines as presented in Table 2. Thus, these results highlight the effectiveness of stacking in enhancing predictive accuracy. Subsequently, Table 3 also presents the final $P3_{SNP-LA}$ pipeline, late-integrating SNP and LA data, that achieves the highest performance with a mean AUC of 0.729, demonstrating the clear advantage of combining multifactorial data types for improved predictive performance in asthma treatment.

**Table 3:** Comparison of performance metrics across the stacked pipelines.

Metric	$P3_{SNP}$	$P3_{LA}$	$P3_{SNP-LA}$
AUC	0.693 ± 0.066	0.625 ± 0.103	0.729 ± 0.048
Weighted precision	0.652 ± 0.047	0.578 ± 0.103	0.701 ± 0.084
Weighted recall	0.642 ± 0.039	0.575 ± 0.099	0.691 ± 0.074
Weighted specificity	0.638 ± 0.037	0.575 ± 0.096	0.688 ± 0.075
Balanced accuracy	0.640 ± 0.039	0.575 ± 0.097	0.689 ± 0.074

Values after the plus-minus sign represent SDs

**Fig. 4:**
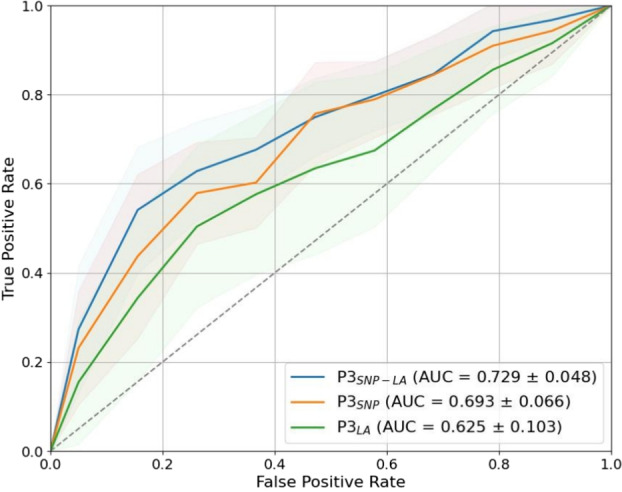
The AUC curves along with the respective SDs for the $P3_{SNP}$, $P3_{LA}$ and $P3_{SNP-LA}$ pipelines, constructed by averaging interpolated true positive rate and false positive rate across 10 folds for each model.

Figure 4 shows that, in contrast to $P3_{SNP}$, the final stacked pipeline, $P3_{SNP-LA}$, which adds LA features to SNP features by stacking $P3_{SNP}$ and $P3_{LA}$, achieves the highest overall performance. While the curves are constructed by averaging interpolated true positive rate and false positive rate across 10 folds and therefore smoothen variability, the fold-wise AUC values capture improvements where $P3_{SNP-LA}$ outperforms $P3_{SNP}$. This is reflected in Table 3 which shows $P3_{SNP-LA}$ yields a mean AUC of 0.729 ± 0.048 (paired *t*-test *p*-value = 0.005), significantly enhancing predictive performance. The weighted precision, weighted recall, and weighted specificity are also improved, achieving values of 0.701 ± 0.084 (paired *t*-test *p*-value = 0.096), 0.691 ± 0.074 (paired *t*-test *p*-value = 0.050), and 0.688 ± 0.075 (paired *t*-test *p*-value = 0.044), respectively.

**Fig. 5:**
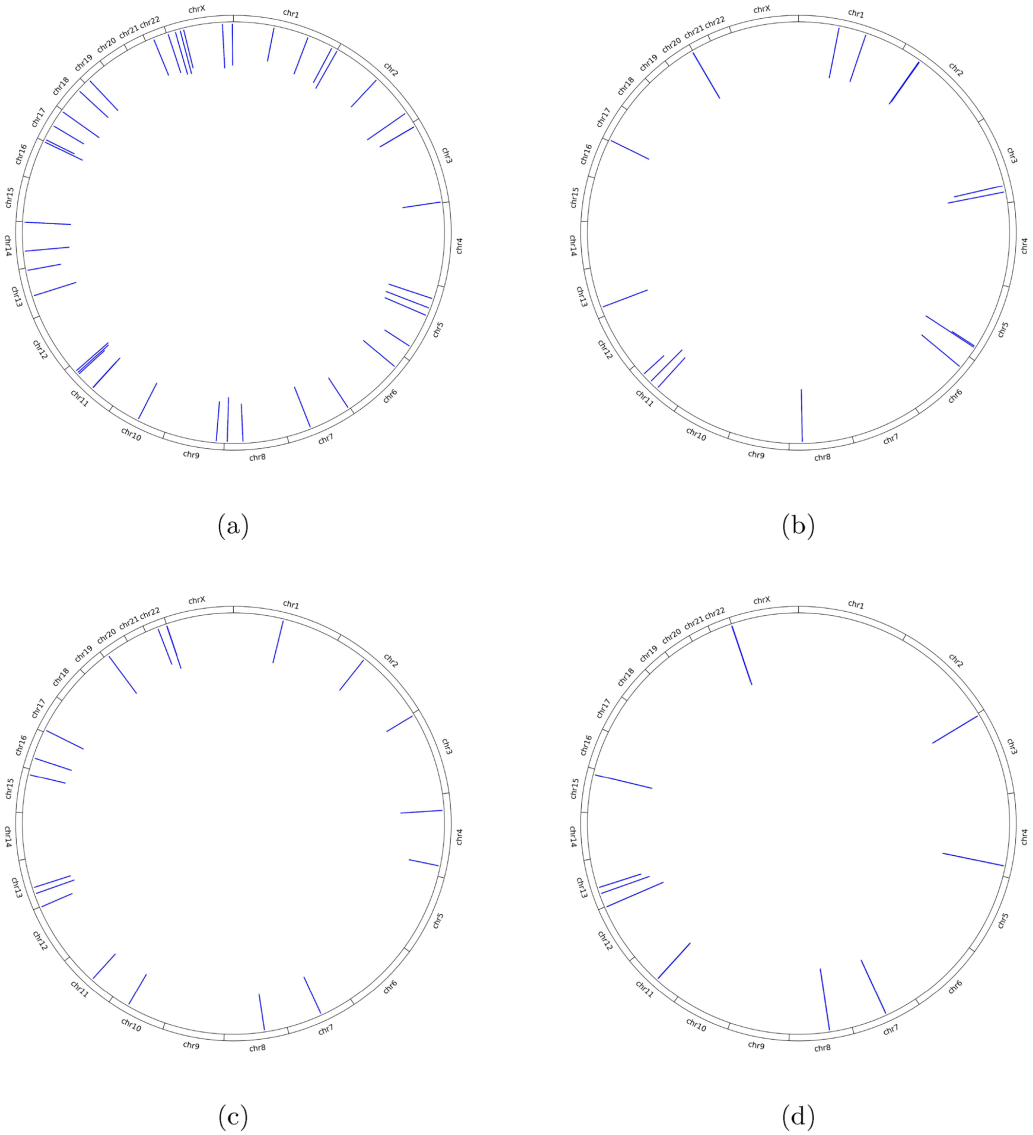
A comparison of the most important loci across chromosomes for each analysis pipeline: (**a**) $P1_{SNP}$, (**b**) $P2_{SNP}$, (**c**) $P1_{LA}$, and (**d**) $P2_{LA}$. The height of each bar represents the SHAP importance values transformed by $\log _{10}(\text {SHAP value}) \times 10$ where taller bars indicate loci with higher predictive importance.

Finally, we run SHAP on the $P1_{SNP}$, $P1_{LA}$, $P2_{SNP}$, and $P2_{LA}$ pipelines to better interpret our results. Figure 5 illustrates the average SHAP values, presented as color intensity, for most important loci across chromosomes for the SNP and LA pipelines. As seen in Fig. 5a and b, the higher SHAP values for the SNP pipelines are on chromosomes 5, 11, and 17, highlighting important genomic regions contributing to the SNP pipelines’ predictions. In contrast, Fig. 5c and d shows the brightest bars for the LA pipelines on chromosomes 3, 11, 13, and 22, indicating strong influence from these regions for LA pipelines’ predictions. The most important loci with the highest average SHAP values (> 0.001) for the pipelines are listed in the Supplementary Tables 1-4. Note that the SNP pipelines have five loci (out of the 59 most important identified) in common and the LA pipelines have 10 loci (out of the 64 most important identified) in common. Further analyzing the most important loci reveals that the SNP and LA pipelines have non-overlapping most important loci, except one locus on chromosome 11 (chr11_62401264_G_A) which is in the most important loci for $P1_{SNP}$, $P2_{SNP}$, and $P2_{LA}$ pipelines.

## Discussion

In this study, we leverage ML techniques to analyze SNP and LA data for predicting SA responses to ICS. Our results show that combining linear models, such as Lasso model, with ensemble non-linear models such as RF significantly enhances the predictive power of our pipelines. The linear model effectively captures simple, additive relationships between variables, while the ensemble RF model is adept at detecting complex, non-linear interactions. This complementary approach maximizes the strengths of each method, leading to a more robust and accurate prediction of treatment response. Additionally, to the best of our knowledge, this study is the first to use ML to integrate LA with genotype data, an underexplored approach in predicting treatment outcomes in complex diseases. Therefore, this approach not only enhances predictive accuracy but also provides a new framework for exploring the genotype and ancestral determinants of treatment response in other diseases.

Our results emphasize the three main contributions of this study. First, we develop models using SNP data and identify the corresponding loci that effectively differentiate ICS responders from non-responders (Table 3, Fig. 5a and b), thereby providing a foundation for personalized asthma treatment. Second, we uncover loci as LA signals (Table 3, Fig. 5c and d) that similarly distinguish responders from non-responders, adding a multifaceted dimension to personalized treatment strategies. Interestingly, our results show that pipelines using LA data alone provide comparable performance to those using SNP data alone (Tables 2 and 3), demonstrating that these distinct data types provide non-overlapping, complementary insights (Fig. 5a–d). Finally, by employing a stacking approach, we achieve a statistically significant improvement in AUC (Table 3 and Fig. 4), highlighting the value of integrating multifactorial data using stacked pipelines to enhance predictive performance.

The addition of LA pipeline to the SNP pipelines notably improves predictive performance. This is aligned with recent studies calling for the integration of LA in genetic research^26,59^. This improvement in results may be associated with the pipelines now adjusted better with genetic context. However, it may also be due to the possibility that the pipelines can now capture gene-environmental interactions and social factors, such as socioeconomic status and access to healthcare, associated with LA. Additionally, our approach may have favored $P3_{SNP}$ more than $P3_{LA}$ because genotype SNPs show direct, pathway-level effects on ICS response^70,71^. By contrast, LA is designed for tract-level discovery in admixed cohorts and, after genotype SNPs are included, LA’s main role is adjustment to control confounding and aid fine-mapping rather than adding large incremental predictive signal^72,73^. Even so, we achieve a significant AUC boost by incorporating LA with SNP data using ML, underscoring the potential of LA-informed ML framework to refine personalized treatment strategies. Additionally, the non-overlapping signals identified in the SNP and LA pipelines (Fig. 5a–d) suggest that different loci may be contributing to the ICS response in ways that are specific to the genotype or ancestral background. Note that not all signals identified are non-overlapping. The overlapping signals, particularly the locus on chromosome 11 that is shared across multiple pipelines, may indicate a strong genetic determinant that is consistent across different genetic backgrounds and modeling approaches. This implies that a multifactorial approach is necessary to fully understand the genetic architecture of asthma and its treatment; thus, it highlights the need for integrated pipelines that can capture this complexity.

This study is subject to several limitations that need to be addressed as part of future work. The small dataset size limits the generalizability of our findings and increases the risk of overfitting; expanding the dataset with additional patient cohorts would enhance the robustness of the pipelines. Second, the absence of external validation further limits the applicability of our results; future work should incorporate independent cohorts for validation to assess the proposed techniques’ generalizability across different populations and diseases. Third, the inability to track medication compliance among patients could introduce bias, as non-compliance may have been misinterpreted as treatment failure. Incorporating medication adherence tracking in future studies would provide a more accurate assessment of treatment outcomes. Additionally, this study provides a foundation for future research where genotype, LA and socio-economic variables can be jointly considered to explore how the use of LA compares with social drivers of health. Lastly, this study does not account for variations in medication composition, which could influence treatment outcomes; adjusting the experiment for medication brands would improve the comprehensiveness of the study.

## Conclusion

This study demonstrates the power of ML in integrating SNP and LA data in addressing severe asthma risk. By improving ICS response predictions, our ML-based stacking approach supports the development of personalized, effective treatment strategies tailored to each patient’s genetic and ancestral profile. Our limited cohort size may constrain the generalizability of these findings and introduce potential overfitting; inclusion of additional patient cohorts in future work would likely strengthen the pipelines’ robustness. Even so, the findings strengthen the case for incorporating multifactorial data in personalized medicine and point to promising avenues for future research and clinical practice.

## Supplementary Information

Supplementary Information.

## Data Availability

Whole exome sequencing was carried out for all subjects in our biorepository project that has been funded through internal (non-federal) funding sources within our Institution. As a result, we have not deposited the data in dBGap. However, we have a well developed process that enable investigators to request data. Our consent form indicates, and our institution requires, that all use of our biorepository data by outside investigators be carried out in the context of a fully collaborative research project. To request data access, please contact Robert Davis at rdavis88@uthsc.edu or visit: https://uthsc.edu/cbmi/big/.

## Declarations

### Approval for human experiments

The research protocol was approved by UTHSC Institutional Review Board (IRB # 22-09164-NHSR) and all research was carried out in accordance with relevant guidelines and regulations. Informed consent was obtained from all participants and/or their legal guardians, and all research was carried out in accordance with the Declaration of Helsinki.
