# Supplementary material for: Machine learning models incorporating genotype and ancestry improve severe asthma risk prediction
Source: Sci Rep. 2025 Nov 17;15:40243. doi: 10.1038/s41598-025-24080-x (PMC12623762; doi:10.1038/s41598-025-24080-x)
Supplement: Supplementary file 1 — Supplementary Information. [file 41598_2025_24080_MOESM1_ESM.pdf]

Machine Learning Models Incorporating Genotype and Ancestry Improve Severe Asthma Risk Prediction

Nahian Tahmin[1], Lokesh K Chinthala[2], Franco Leonel Marsico[3], Silvia Buonaiuto[3], Akram Mohammed[2], Annette Carlisle[2], Yadu Gautam[4], Vincenza Colonna[3], Tesfaye B. Mersha[4], Robert L Davis[2], Anahita Khojandi[5*]

[1]Bredesen Center of Interdisciplinary Research, University of Tennessee, Knoxville, Cumberland Ave, Knoxville, 37996, TN, USA.
[2]Department of Pediatrics, University of Tennessee Health Science Center, Monroe Avenue, Memphis, 38163, TN, USA.
[3]Department of Genetics, Genomics and Informatics , University of Tennessee Health Science Center, Monroe Avenue, Memphis, 38163, TN, USA.
[4]Department of Medicine, Indiana University School of Medicine, Walnut Street, Indianapolis, 46202, IN, USA.
[5*]Department of Industrial and Systems Engineering, University of Tennessee, Knoxville, Cumberland Ave, Knoxville, 37996, TN, USA.

*Corresponding author(s). E-mail(s): khojandi@utk.edu;
Contributing authors: ntahmin@vols.utk.edu; lchintha@uthsc.edu; fmarsic1@uthsc.edu ; sbuonaiu@uthsc.edu; amoham18@uthsc.edu; acarlis3@uthsc.edu ; ygautam@iu.edu; vcolonna@uthsc.edu; tmersha@iu.edu ; rdavis88@uthsc.edu;

Supplementary Table 1: The most important loci from $P1_{SNP}$ based on averaged SHAP values (>0.001) that are the present in at least eight or more CV folds.

Gene	SNP	Mean SHAP Value
GALNT10, SAP30L-AS1	chr5_154329713_A_G	0.048850
ZNF594-DT, SCIMP	chr17_5214838_TA_T	0.039347
MYO16	chr13_108898213_A_G	0.031213
ABCA4	chr1_94010999_G_A	0.029293
CWF19L2	chr11_107326880_G_C	0.028010
ASRGL1, LOC107984335, SCGB1A1	chr11_62401264_G_A	0.027186
KRT9	chr17_41570024_C_A	0.027080
SDK1, LOC105375131	chr7_4129429_A_G	0.025206
PAX8, PAX8-AS1	chr2_113242159_G_T	0.022366
IRAK2	chr3_10209512_A_G	0.021104
UBR5	chr8_102327653_A_C	0.020273
MUC4	chr3_195780043_G_A	0.019369
TMEM191B, PI4KAP1, Metazoa_SRP	chr22_18529904_C_A	0.018379
NEDD4L	chr18_58387384_AT_A	0.018375
GNG4	chr1_235552317_A_C	0.018005
TNR	chr1_175355515_A_G	0.017518
BCOR	chrX_40075235_C_T	0.017141
IL17RC	chr3_9928227_C_T	0.016786
HAUS6	chr9_19058427_G_A	0.016443
TMEM258, MYRF	chr11_61776027_T_C	0.015799
XG	chrX_2797384_A_G	0.015371
MYPN	chr10_68174501_C_G	0.015292
MUC22	chr6_31025411_A_C	0.015270
ZNF439, ZNF69	chr19_11887962_C_T	0.014664
POU2AF1	chr11_111377789_C_G	0.014146
TREX2, HAUS7	chrX_153462697_T_C	0.013665
CHMP1A	chr16_89648370_C_T	0.013463
BUD13, ZPR1	chr11_116770312_C_T	0.012430
LOC101927262, ALKAL2, LOC105373346	chr2_293719_A_G	0.011757
SH2B2, CUX1	chr7_102285204_C_G	0.011353
SLC25A14	chrX_130371787_C_T	0.010443
RPS6KA3	chrX_20187738_A_C	0.010399
RB1, RCBTB2	chr13_48496376_G_C	0.010353
SEC23A	chr14_39074610_T_TAAG	0.010163
COL22A1	chr8_138663839_A_G	0.010111
DMD	chrX_31836665_G_C	0.010101
ARHGEF28	chr5_73901360_A_C	0.010058
RNF213-AS1, RNF213	chr17_80363258_G_C	0.010019
NPR3	chr5_32716368_C_CT	0.008756
SLC38A9	chr5_55656860_C_CTT	0.008663
ATIC	chr2_215325931_C_T	0.008272
LOC107984634, IGHVIII-2-1, IGHV1-3	chr14_106005385_G_A	0.008270

Supplementary Table 2: The most important loci from $P1_{LA}$ based on averaged SHAP values (>0.001) that are the present in at least eight or more CV folds.

**Gene**	**SNP**	**Mean SHAP Value**
TLL1	chr4_165994329_A_T	0.045111
SUMF1, MRPS10P2	chr3_4411053_G_A	0.043824
CHKB, MAPK8IP2	chr22_50603234_C_A	0.037819
INCENP	chr11_62128069_A_G	0.032382
GALNT9, LOC100130238	chr12_132272953_G_A	0.031172
PDE6C	chr10_93612977_G_A	0.026778
RNU7-195P, ABHD2	chr15_89116236_C_G	0.023449
ALKAL1	chr8_52534696_C_A	0.023164
HTR2A	chr13_46892646_T_G	0.019470
ZNRF3	chr22_29044704_A_C	0.019396
HMGA1P8, WDR54, RTKN, C2orf81	chr2_74422789_TC_T	0.018808
KDM8	chr16_27204059_A_C	0.018548
FRY	chr13_32225099_A_G	0.015576
POR, TMEM120A	chr7_75989109_T_G	0.015175
WSCD1	chr17_6110856_A_G	0.013947
RELL1, C4orf19	chr4_37588948_G_A	0.013217
VANGL1	chr1_115663802_G_A	0.011331
TTLL8	chr22_50030478_C_T	0.009532
ANKEF1, SNAP25-AS1	chr20_10044303_G_T	0.007938

Supplementary Table 3: The most important loci from $P2_{SNP}$ based on averaged SHAP values (>0.001) that are the present in at least eight or more CV folds.

**Gene**	**SNP**	**Mean SHAP Value**
GALNT10, SAP30L-AS1	chr5_154329713_A_G	0.064260
CWF19L2	chr11_107326880_G_C	0.064216
ASRGL1, LOC107984335, SCGB1A1	chr11_62401264_G_A	0.015463
TRPV3	chr17_3542607_T_G	0.012537
DLG2	chr11_83980458_A_G	0.010558
SKA3	chr13_21155813_T_TA	0.007161
MSH6, FBXO11	chr2_47803699_A_T	0.006578
MUC22	chr6_31025411_A_C	0.006000
FCRL1	chr1_157798210_G_C	0.005892
PTX3, VEPH1	chr3_157437072_A_G	0.005836
ABCA4	chr1_94010999_G_A	0.005031
EPAS1	chr2_46356142_C_T	0.004714
COL9A3, OGFR	chr20_62817581_C_A	0.004514
TRPS1	chr8_115623715_C_T	0.004382
LOC105372709, CABLES2, RBBP8NL	chr20_62410854_G_A	0.004026
FNDC3B	chr3_172330804_A_G	0.002675
ADAM19, NIPAL4	chr5_157471682_A_G	0.002605

Supplementary Table 4: The most important loci from $P2_{LA}$ based on averaged SHAP values (>0.001) that are the present in at least eight or more CV folds.

Gene	SNP	Mean SHAP Value
HTR2A	chr13_46895805_G_A	0.010931
HTR2A	chr13_46892646_T_G	0.010573
SCGB1D2	chr11_62243391_C_T	0.008340
INCENP	chr11_62145619_G_C	0.008195
INCENP	chr11_62148840_C_T	0.008134
LOC102723765, SCGB1A1	chr11_62419070_G_A	0.007960
ASRGL1	chr11_62337919_C_T	0.007912
INCENP	chr11_62129937_C_T	0.007797
SCGB2A1	chr11_62210386_CTTTTTTT_C	0.007732
INCENP	chr11_62140159_T_A	0.007391
ASRGL1, LOC107984335, SCGB1A1	chr11_62401264_G_A	0.007325
INCENP	chr11_62128069_A_G	0.007266
ASRGL1	chr11_62357224_C_CTTTTG	0.007211
ASRGL1	chr11_62356245_G_A	0.007084
SCGB2A2	chr11_62271280_T_A	0.007031
SCGB1D4	chr11_62296427_C_CA	0.006919
AHNAK	chr11_62433986_A_G	0.006792
FRY	chr13_32225099_A_G	0.004950
SUMF1	chr3_4467058_C_T	0.004809
ITPR1	chr3_4516420_T_C	0.004683
SUMF1, MRPS10P2	chr3_4411053_G_A	0.004443
ITPR1	chr3_4520983_T_G	0.004273
SUMF1	chr3_4420014_C_T	0.004185
ABHD2	chr15_89155307_A_G	0.002360
SRRM3	chr7_76235145_T_G	0.002340
POR, TMEM120A	chr7_75989109_T_G	0.002292
RNU7-195P, ABHD2	chr15_89116236_C_G	0.002274
TTLL8	chr22_50030478_C_T	0.002186
SRRM3	chr7_76259866_T_C	0.002160
RB1CC1	chr8_52674146_A_G	0.002032
GALNT9	chr12_132286390_G_A	0.001898
GALNT9	chr12_132286509_CT_C	0.001875
ALKAL1	chr8_52539799_T_TA	0.001875
RB1CC1	chr8_52642661_C_T	0.001804
TLL1	chr4_166060256_T_TA	0.001766
RB1CC1	chr8_52623916_T_TCA	0.001708
ARSA	chr22_50625713_G_A	0.001669
SHANK3	chr22_50704264_T_A	0.001643
TLL1	chr4_166099626_C_CA	0.001639
TLL1	chr4_165994329_A_T	0.001613
GALNT9, LOC100130238	chr12_132272953_G_A	0.001606
RNA5SP170, TLL1	chr4_166057387_CT_C	0.001576
SHANK3	chr22_50704881_G_C	0.001539
ACR	chr22_50744827_A_G	0.001530
ALKAL1	chr8_52534696_C_A	0.001519
